# Does weight mediate the effect of smoking on coronary heart disease? Parametric mediational g-formula analysis

**DOI:** 10.1371/journal.pone.0262403

**Published:** 2022-01-13

**Authors:** Yaser Mokhayeri, Maryam Nazemipour, Mohammad Ali Mansournia, Ashley I. Naimi, Jay S. Kaufman

**Affiliations:** 1 Cardiovascular Research Center, Shahid Rahimi Hospital, Lorestan University of Medical Sciences, Khorramabad, Iran; 2 Osteoporosis Research Center, Endocrinology and Metabolism Clinical Sciences Institute, Tehran University of Medical Sciences, Tehran, Iran; 3 Department of Epidemiology and Biostatistics, School of Public Health, Tehran University of Medical Sciences, Tehran, Iran; 4 Department of Epidemiology, Emory University, Atlanta, GA, United States of America; 5 Department of Epidemiology, Biostatistics and Occupational Health, Faculty of Medicine, McGill University, Montreal, Quebec, Canada; German Centre for Neurodegenerative Diseases Site Munich: Deutsches Zentrum fur Neurodegenerative Erkrankungen Standort Munchen, GERMANY

## Abstract

**Background:**

In settings in which there are time-varying confounders affected by previous exposure and a time-varying mediator, natural direct and indirect effects cannot generally be estimated unbiasedly. In the present study, we estimate interventional direct effect and interventional indirect effect of cigarette smoking as a time-varying exposure on coronary heart disease while considering body weight as a time-varying mediator.

**Methods:**

To address this problem, the parametric mediational g-formula was proposed to estimate interventional direct effect and interventional indirect effect. We used data from the Multi-Ethnic Study of Atherosclerosis to estimate effect of cigarette smoking on coronary heart disease, considering body weight as time-varying mediator.

**Results:**

Over a 11-years period, smoking 20 cigarettes per day compared to no smoking directly (not through weight) increased risk of coronary heart disease by an absolute difference of 1.91% (95% CI: 0.49%, 4.14%), and indirectly decreased coronary heart disease risk by -0.02% (95% CI: -0.05%, 0.04%) via change in weight. The total effect was estimated as an absolute 1.89% increase (95% CI: 0.49%, 4.13%).

**Conclusion:**

The overall absolute impact of smoking to incident coronary heart disease is modest, and we did not discern any important contribution to this effect relayed through changes to bodyweight. In fact, changes in weight because of smoking have no meaningful mediating effect on CHD risk.

## Introduction

Coronary heart disease (CHD), also known as coronary artery disease and ischemic heart disease, is the leading cause of death in the world [1]. Although, mean percentage change in number of Years of Life Lost (YLL) from 2007 to 2017 due to CHD has increased by 17.3%, mean percentage change in age-standardized YLL rate has decreased by -9.8% in the same period [2].

Exposure to smoking between 1990 and 2015 declined worldwide by 25%; nevertheless, it still ranked among the leading five risk factors for attributable disability-adjusted life year (DALYs). In high-income countries such as the US, Canada, and the UK, smoking is considered the most important risk factor for attributable DALYs for both sexes [3].

LDL cholesterol, HDL cholesterol, triglyceride, body mass index, glucose, and blood pressure may mediate the relationship between smoking and CHD [4]. In smokers compared to nonsmokers, body mass indices are lower [5–7]. Chen et al. (2019), reported that smoking cessation could reduce 10-year risk of CHD, however, gaining weight following smoking cessation could conceal to a small extent the beneficial effect of the quitting [8]. Tamura et al. (2010) concluded that in spite of gaining weight after smoking cessation, the total risk of CHD was decreased in men [9]. Luo et al. (2013) according to their study on women, indicated that the relationship between smoking cessation and incidence of CHD could be weakened by gaining weight [10]. None of these studies appropriately adjusted for time-varying confounders using cohort data with repeated exposure and mediators [11–13]. G-methods (generalized methods) as causal methods could appropriately adjust for time-varying confounders affected by the prior exposure [14–18]. In frameworks with longitudinal data, intermediate confounder could not be adjusted using standard analytic approaches [19]. The intermediate confounder, which is likely not rare in mediation analysis, is both a mediator-outcome confounder and a variable in the causal direct path [19]. Moreover, to identify natural direct effect (NDE) and natural indirect effect (NIE) four assumptions are needed namely 1- no unmeasured exposure-outcome confounder 2- no- unmeasured mediator-outcome confounder 3- no unmeasured exposure-mediator confounder, and 4- no mediator-outcome confounder affected by the prior exposure [20]. In the presence of intermediate confounder, the fourth assumption would be violated, consequently natural direct and indirect effect are not identified. Addressing partially this problem, VanderWeele and Tchetgen Tchetgen using the mediational g-formula sought to find a solution and proposed the pure interventional direct effect (IDE) also known as a random interventional analogue of the natural direct effect and total interventional indirect effect (IIE) [21]. As VanderWeels and Tchetgen Tcheten’s method was semiparametric, Lin et al developed a fully parametric mediational g-formula [22].

In the present study, we estimate IDE and IIE of cigarette smoking—as a time-varying exposure—on CHD while considering weight as both a time-varying mediator of past cigarette smoking, and a time-varying confounder of future cigarette smoking, using the parametric mediational g-formula to control for time-varying confounding. We estimate the direct (not through weight) and indirect (through weight) effects in data obtained from the Multi-Ethnic Study of Atherosclerosis (MESA).

## Methods

### Study population

We used the Multi-Ethnic Study of Atherosclerosis (MESA) data, a community-based cohort of adults, started in 2000 on 6814 men and women aged 45 to 84 years. Some additional details of the study have been presented elsewhere [23].

Five participants were excluded from our analysis because they already had prevalent CVD diagnoses at baseline. At the baseline, there were 6809 participants. It should be noted, since mGFORMULA SAS macro does not handle missing data, the final analysis limited to those participants without death or loss to follow up during the period of study (4433 participants). The participants were followed for more than eleven years and five visits. The first visit was scheduled for July 15, 2000 through July 14, 2002 (24 months); the second visit, scheduled for July 15, 2002 through January 14, 2004 (18 months); the third visit, scheduled for January 15, 2004 through July 14, 2005 (18 months); the fourth visit, scheduled for July 15, 2005 through July 14, 2007 (24 months); and the fifth visit, scheduled for April 1, 2010 through September 30, 2011 (18 months).

### Exposure, mediator, outcome, and confounders

Average number of cigarettes smoked per day—as the exposure—was self-reported. The data were obtained via questionnaire. Participants were asked if they smoked during last 30 days, those who replied affirmatively were asked for report the average number of cigarettes smoked per day. In this study, we aimed to estimate the causal effect of ‘had everyone been a smoker/smoked 20 cigarettes per day’ vs. ‘had everyone been a non-smoker/smoked 0 cigarettes per day’, over the course of follow-up, on incident CHD or death from CHD. We defined weight in kilogram (*kg*) as the mediator. Weight was assessed using a standard weighing scale to the nearest 0.5 *kg*. CHD events as the outcome included myocardial infarction, resuscitated cardiac arrest, definite angina, probable angina (if followed by revascularization), and CHD death. Questionnaires, interviews, and medical records were used to obtain the date of CHD to the nearest day. Time-varying covariates measured at all visits including intentional physical activity, total cholesterol, hypertension, hypertension medication, and current aspirin use entered in the models as potential confounders. Additionally, we also adjusted for baseline age, sex, race/ethnicity (White, Asian, Hispanic, and African-American), alcohol consumption (drinks per week), and diet score [24], baseline smoking (never, former, and current smoker), annual family income, education level. A standard and validated questionnaire was used to obtain data on recreational physical activity. Valid test kits were used to obtain data on total cholesterol. An appropriately sized cuff was used to obtain resting blood pressure from the right arm after five minutes in the seated position. Three readings were taken; the average of the second and third readings were used as the blood pressure levels in the study.

### Statistical analysis

If we describe *mediation* as a two-stage process: 1- *M-stage* which denotes the process that cause the mediator and 2- *Y-stage*, a process that cause the outcome; hence, four potential outcomes would be obtained. Y0M0 is the outcome that would be observed if an individual were unexposed and had a mediator if she/he would have if unexposed (Y0M0 = Y0). Y1M1 is the outcome that would be observed if an individual were exposed and had a mediator if she/he would have if exposed (Y1M1 = Y1). Y1M0 is the outcome that would be observed if an individual were exposed but had a mediator if she/he would have if unexposed. Y0M1 is the outcome that would be observed if an individual were unexposed but had a mediator if she/he would have if exposed. Using the parametric mediational g-formula, the *M-stage* is a random draw form the distribution of the mediator among those with exposure status *A = a*. Therefore, it would have fixed the mediator to the level that is randomly chosen from the distribution of the mediator among those with exposure status. Mediational g-formula is related to both Robins’ regular g-formula [12] and Pearl’s mediation formula [25]. In fact, in the absence of mediation it reduces to g-formula and in the absence of time-varying confounders reduces to mediation formula. In defining outcomes this way, we are able to quantify outcome risks under several different exposure and mediator scenarios such that IDE and IIE could be estimated. We estimated (*ā*_1_, *ā*_2_) for each (*ā*_1_, *ā*_2_) = (*ā*, *ā*), (*ā*, ā^*^), (ā^*^, *ā)*, (ā^*^, ā^*^). These measures indicate the simulated risk of CHD. This way, pure IDE, total IIE, and interventional analogue of total effect (ITE) can be defined as Eqs 1, 2, and 3, respectively.


$$\mathrm{IDE}=[Q(\bar{a},\;{\bar{a}}^{*})‐Q({\bar{a}}^{*},\;{\bar{a}}^{*})]=[P(Y_{1M_{0}})‐P(Y_{0M_{0}})]$$ (1)

$$IIE=[Q(\bar{a},\;\bar{a})‐Q(\bar{a},\;{\bar{a}}^{*})]=[P(Y_{1M_{1}})‐P(Y_{1M_{0}})]$$ (2)

$$ITE=[Q(\bar{a},\;\bar{a})‐Q({\bar{a}}^{*},\;{\bar{a}}^{*})]=[P(Y_{1M_{1}})‐P(Y_{0M_{0}})]$$ (3)

**Fig 1:**
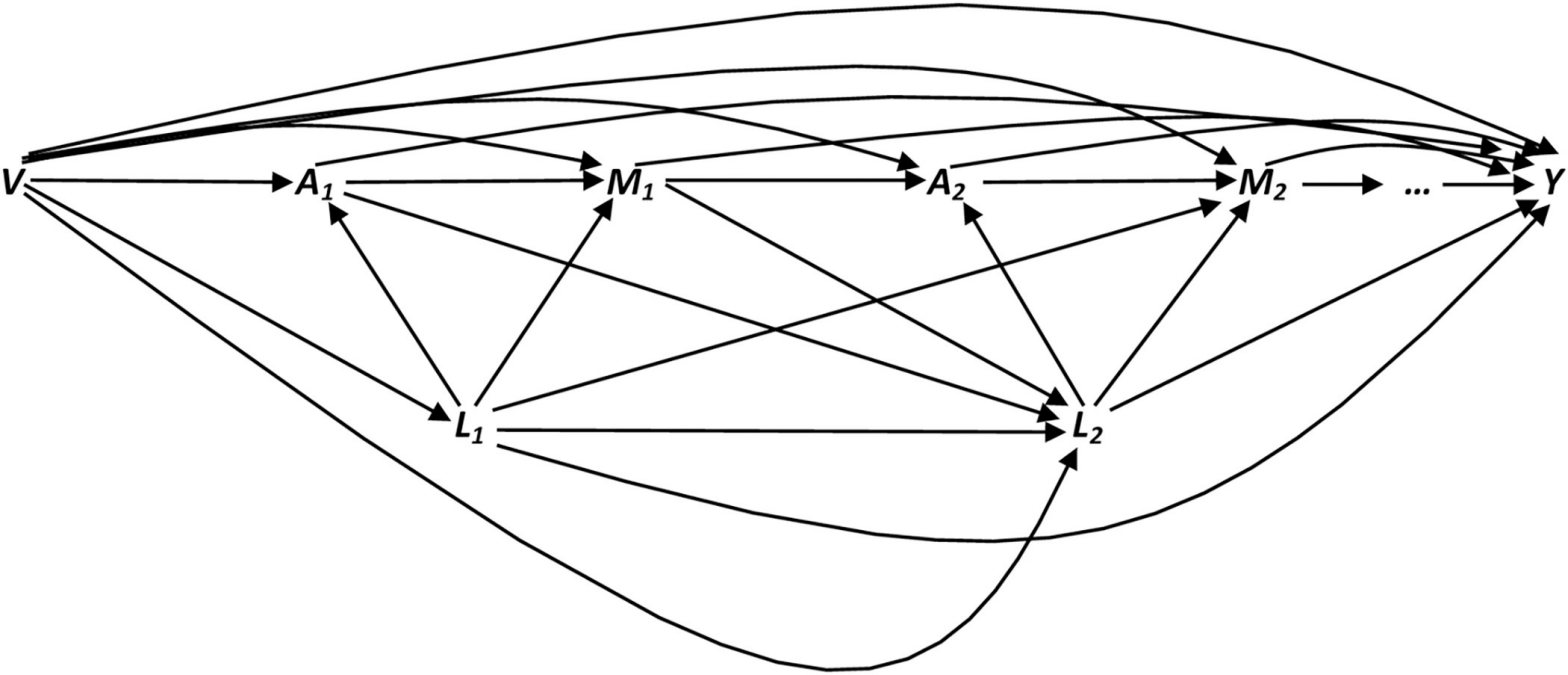
Causal diagram depicting the effect of time-varying exposure (smoking) on the outcome (CHD) in the presence of time-varying mediator (weight). V, A, M, L, and Y stand for baseline confounders, time-varying exposure, time-varying mediator, time-varying confounders, and the outcome, respectively.

Fig 1 is a causal directed acyclic graph [26–32] depicting the relationship between our time-varying exposure (cigarette smoking), time-varying mediator (weight), time-varying confounders, and CHD. *V* denotes baseline confounders (e.g. age); *A* represents the time-varying exposure (cigarette smoking); *M* denotes time-varying mediator (weight); *L* represents the time-varying confounders (e.g. physical activity) which are affected by the prior exposures; and *Y* corresponds to the binary outcome (CHD). Subscripts of 0 and 1 correspond to visits 0 and 1 of the study, respectively (to simplify the graph, just 2 visits were depicted).

We used Monte-Carlo estimation [33] to calculate the point estimates, and the nonparametric bootstrap with 500 resamples to obtain 95% confidence intervals. We used mGFORMULA SAS macro (S1 Appendix).

## Results

After 51,487 person-years of follow-up (median duration of follow-up 8.45 and IQR of 1.01 years) 388 new CHD cases occurred. Of 6809 eligible participants 401 (5.9%) died from causes other than CHD. Moreover, 1987 (29%) of participants were lost to follow-up during the study. Final sample included in the analysis is 4433 participants. Baseline characteristic of the 6,809 eligible participants are illustrated in Table 1. The smokers compared to quitters and nonsmokers were more likely to drink more alcohol, to have less intentional physical activity, to have less education levels, to have less family income, and to have less prevalence of hypertension and hypertension medication. The quitters compared to smokers and nonsmokers were likely to be men and to have more weight. Complete case analysis was performed, as the proportion of covariate missing data was low: For hypertension 2.2%, physical activity 1.5%, total cholesterol 3.1%, hypertension medication 3.2%, and current aspirin 1.6%.

**Table 1:** Baseline characteristics of eligible participants in the Multi-Ethnic Study of Atherosclerosis, United States, 2000–2011.

Characteristics	Smoker (n = 890)	Quitter (n = 2493)	Nonsmoker (n = 3426)
**Age,** y, mean (SD)	58.15 (9.15)	63.48 (9.83)	62.21 (10.52)
**Race/ethnicity,** n (%)			
**Caucasian**	302 (33.93)	1158 (46.45)	1159 (33.83)
**Asian**	45 (5.06)	153 (6.14)	606 (17.69)
**African-American**	340 (38.20)	697 (27.96)	854 (24.93)
**Hispanic**	203 (22.81)	485 (19.45)	807 (23.56)
**Male,** n (%)	468 (52.58)	1440 (57.76)	1302 (38)
**Diet score,** mean (SD)	2.50 (5.10)	4.64 (4.77)	4.61 (4.55)
**Alcohol consumption,** drinks/week, mean (SD)	6.40 (11.32)	5.81 (9.82)	2.11 (5.81)
**Education,** n (%)			
**≤ high school**	350 (39.46)	797 (32.09)	1314 (38.48)
**college–associate degree**	329 (37.09)	761 (30.64)	843 (24.69)
**≥ bachelor degree**	208 (23.45)	926 (37.28)	1258 (36.84)
**Annual family income,** n (%)			
**< $25000**	275 (32.51)	666 (27.91)	1115 (33.75)
**$25,000-$50,000**	277 (32.74)	677 (28.37)	938 (28.39)
**> $50,000**	294 (34.75)	1043 (43.71)	1251 (37.86)
**Hypertension,** n (%)	333 (37.42)	1178 (47.25)	1545 (45.10)
**Hypertension medication,** n (%)	265 (29.78)	979 (39.27)	1291 (37.68)
**Current aspirin use,** n (%)	143 (16.07)	601 (24.11)	651 (19)
**Total cholesterol,** mmol/l, mean (SD)	4.98 (1.01)	4.98 (0.90)	5.06 (0.91)
**Intentional physical activity,** min/week, mean (SD)	351.93 (557.14)	385.23 (547.45)	354.64 (503.80)
**Weight,** kg, mean (SD)	79.58 (16.92)	81.81 (17.06)	75.99 (16.93)

SD: standard deviation.

The results of the standard parametric g-formula in Table 2 shows that the estimated 11-year risk of CHD in ‘had everyone been a smoker/smoked 20 cigarettes per day’ vs. ‘had everyone been a non-smoker/smoked 0 cigarettes per day’ were 6.92% and 5%, respectively. Therefore, risk ratio was estimated as 1.38 (95% CI: 1.04, 1.86). The estimated risk difference using standard parametric g-formula was 1.92%, which is close to the estimated risk difference for CHD—1.89%—using parametric mediational g-formula (Table 3).

**Table 2:** Total effect of smoking 20 cigarettes per day compared to no smoking on CHD, using parametric g-formula in the Multi-Ethnic Study of Atherosclerosis, United States, 2000–2011.

Intervention	11-year Risk, %	95% CI	Risk Difference, %	95% CI
**No intervention**	5.94	3.74, 9.46	0.94	0.03, 2.01
**No smoking**	5.00	2.65, 8.60	Ref.	
**20 cigarettes per day**	6.92	4.00, 11.14	1.92	0.19, 4.61

CHD: coronary heart disease, CI: confidence interval.

**Table 3:** Mediation analysis for the effect of smoking 20 cigarettes per day compared to no smoking on CHD, mediated by weight in the Multi-Ethnic Study of Atherosclerosis, United States, 2000–2011.

	Risk Difference, %	95% CI
**Interventional total effect**	1.89	0.49, 4.13
**Interventional direct effect**	1.91	0.49, 4.14
**Interventional indirect effect**	-0.02	-0.05, 0.04
**E** (Y0M0)	5.03	2.90, 7.95
**E** (Y1M0)	6.94	4.01, 10.3
**E** (Y0M1)	4.89	2.90, 7.91
**E** (Y1M1)	6.98	4.01, 10.05

CHD: coronary heart disease, CI: confidence interval.

In Table 3, using the estimates of the joint exposure and mediator interventions—simulated risks, we calculated interventional total effect, direct effect, and indirect effect (through weight) of smoking on CHD. Using parametric mediational g-formula we simulated CHD risks under no smoking with weight distributed as the weight under no smoking: *P(Y0M0)*; smoking 20 cigarettes per day with weight distributed as the weight under no smoking: *P(Y1M0)*; no smoking with weight distributed as the weight under smoking 20 cigarettes per day: *P(Y0M1)*; and smoking 20 cigarettes per day with weight distributed as the weight under smoking 20 cigarettes per day: *P(Y1M1)*. The results are presented on the risk difference scale. We estimated that smoking could directly (not through weight) increase CHD by 1.91% (95% CI: 0.49%, 4.14%), and decrease CHD as -0.02% (95% CI: -0.05%, 0.04%) via change in weight.

## Discussion

We found that smoking might have either a very small or no indirect effect on CHD through weight. Clair et al. (2013), testing the hypothesis that weight gain following smoking cessation does not attenuate the benefits of smoking cessation among adults with and without diabetes, indicated that weight gain after smoking cessation does not modify the association between smoking and risk of CVD events [34].

Feodoroff et al. investigating the dose-dependent effect of smoking on CHD in type 1 diabetes, indicated that smoking one pack per day (20 cigarettes) compared with never smokers adjusted for age, sex, BMI, hypertension, duration of diabetes, and HbA_1c_ could increase the risk of incidence of CHD (HR = 1.45, 95% CI: 1.15, 1.84) [35]. Doyle et al. concluded that risk ratio for smoking 15–35 cigarettes per day compared to non-smokers in men is 1.6 [36]. Woodward et al. reported that men who smoke ≥20 cigarette per day compared to non-smokers for CHD have a hazard ratio 1.93 (95% CI: 1.15, 3.24). This value for women estimated as 3.81 (95% CI: 2.00, 7.27) [37]. Mucha et al. conducted a meta-analysis study and indicated that a low-level use of smoking (≤20 cigarettes per day) results in a risk ratio as 1.70 (95% CI: 1.52, 1.90) and a high-level use (˃20 cigarettes per day) could cause a risk ratio of 2.09 (95% CI: 1.87, 2.34) [38]. Munafò et al. (2009), observed that at the baseline of a longitudinal study of men, never smokers and ex-smokers have a higher BMI (at average 1.6 kg/m2) compared to current smokers. The results were not changed after adjusting for age, socioeconomic position, alcohol, and calorie consumption. They also indicated an increase of 1.56 kg/m2 (95% CI: 1.29, 1.82) after smoking cessation adjusted for age, socioeconomic position, alcohol, and calorie consumption [5]. Sneve et al. using data of the Tromsø study resulted that current smokers in both genders compared to never- smokers have a lower BMI. They also reported that smoking cessation is related to an increase in weight [6].

The validity of the results depends on assumptions of positivity and consistency. The positivity assumption implies that the strata created by all confounder and treatment levels (exposed and unexposed) must be observed. However, the parametric g formula is less prone to bias induced by positivity violations. Consistency implies that the outcome for every exposed or unexposed individual (observed outcome) is equal with the outcome if they had received exposure or remained unexposed (counterfactual outcome), respectively. We note that similarity between observed and estimated risk is a necessary but insufficient condition for the assumption of no misspecification. In our study, the simulated 11-year risk of CHD under no intervention was 5.94% and the observed risk was 6.91%. As a strength of our analysis, the parametric mediational g-formula [22] is a valid statistical strategy to estimate the direct and indirect effects of time-varying exposure on incidence of CHD with time-varying mediator (weight). Using this statistical method, we could appropriately adjust for a variety of time-varying confounders, which are affected by prior exposures. As VanderWeele and Tchetgen Tchetgen’s approach was semiparametric, we used a fully parametric mediational g-formula developed by Lin et al. (2017).

Our study is subject to some limitations. First, the validity of the estimated effects relies on the assumption of no unmeasured confounding for the effects of exposure on outcome, exposure on mediator and mediator on outcome, no measurement error, and correct specification of the parametric models used in the analysis. However, there was measurement error in self-reported cigarette smoking due to recall and underreporting biases cigarette smoking [39]. Physical activity was not assessed in visit 4, so we carried forward the values at visit 3. Moreover, we have not included occupational physical activity in the model. The presence of measurement error in confounders like physical activity and alcohol consumption will lead to residual confounding [40]. Second, the parametric mediational g-formula could not be used for multiple mediators. In fact, using this approach, we can just estimate one single direct/indirect effect. Third, the parametric-formula is also subject to the g-null paradox—it could lead to rejecting the causal null, even when it is true [41]. However, it could not be a concern in our setting as the effect of cigarette smoking and weight on CHD has been shown previously. Fourth, missing data, (censoring and competing risk) may result in selection bias but there is no function in the macro of mediational g-formula for handling missing data yet.

For future studies, Schomaker and Heumann (2018) regarding some estimators, which require bootstrapping to estimate confidence intervals, presented four methods to combine bootstrap estimation with multiple imputation. They indicated that the proportion of missingness and the number of multiple imputed data sets affect methods performance [42].

## Conclusion

The overall absolute impact of smoking to incident coronary heart disease is modest, and we did not discern any important contribution to this effect relayed through changes to bodyweight. In fact, changes in weight because of smoking have no meaningful mediating effect on CHD risk.

## Supporting information

S1 Appendix (PDF)

## References

[1] Roth GA, Johnson C, Abajobir A, Abd-Allah F, Abera SF, Abyu G, et al. Global, regional, and national burden of cardiovascular diseases for 10 causes, 1990 to 2015. Journal of the American College of Cardiology. 2017;70(1):1–25. doi: 10.1016/j.jacc.2017.04.052 28527533 PMC5491406

[2] Roth GA, Abate D, Abate KH, Abay SM, Abbafati C, Abbasi N, et al. Global, regional, and national age-sex-specific mortality for 282 causes of death in 195 countries and territories, 1980–2017: a systematic analysis for the Global Burden of Disease Study 2017. The Lancet. 2018;392(10159):1736–88. 10.1016/S0140-6736(18)32203-7 PMC6227606 30496103

[3] Forouzanfar MH, Afshin A, Alexander LT, Anderson HR, Bhutta ZA, Biryukov S, et al. Global, regional, and national comparative risk assessment of 79 behavioural, environmental and occupational, and metabolic risks or clusters of risks, 1990–2015: a systematic analysis for the Global Burden of Disease Study 2015. The Lancet. 2016;388(10053):1659–724. doi: 10.1016/S0140-6736(16)31679-8 27733284 PMC5388856

[4] Price J, Mowbray P, Lee A, Rumley A, Lowe G, Fowkes F. Relationship between smoking and cardiovascular risk factors in the development of peripheral arterial disease and coronary artery disease; Edinburgh Artery Study: Edinburgh Artery Study. European heart journal. 1999;20(5):344–53. doi: 10.1053/euhj.1998.1194 10206381

[5] Munafò MR, Tilling K, Ben-Shlomo Y. Smoking status and body mass index: a longitudinal study. Nicotine & Tobacco Research. 2009;11(6):765–71. doi: 10.1093/ntr/ntp062 19443785

[6] Sneve M, Jorde R. Cross-sectional study on the relationship between body mass index and smoking, and longitudinal changes in body mass index in relation to change in smoking status: the Tromsø Study. Scandinavian journal of public health. 2008;36(4):397–407. doi: 10.1177/1403494807088453 18539694

[7] Freathy RM, Kazeem GR, Morris RW, Johnson PC, Paternoster L, Ebrahim S, et al. Genetic variation at CHRNA5-CHRNA3-CHRNB4 interacts with smoking status to influence body mass index. International journal of epidemiology. 2011;40(6):1617–28. doi: 10.1093/ije/dyr077 21593077 PMC3235017

[8] Chen S, Kawasaki Y, Hu H, Kuwahara K, Yamamoto M, Uehara A, et al. Smoking cessation, weight gain, and the trajectory of estimated risk of coronary heart disease: 8-year follow-up from a prospective cohort study. Nicotine & Tobacco Research. 2019. 10.1093/ntr/ntz165 PMC7789946 31504860

[9] Tamura U, Tanaka T, Okamura T, Kadowaki T, Yamato H, Tanaka, H, et al. Changes in weight, cardiovascular risk factors and estimated risk of coronary heart disease following smoking cessation in Japanese male workers: HIPOP-OHP study. Journal of atherosclerosis and thrombosis. 2010:1001150159– doi: 10.5551/jat.1800 20081325

[10] Luo J, Rossouw J, Margolis KL. Smoking cessation, weight change, and coronary heart disease among postmenopausal women with and without diabetes. Jama. 2013;310(1):94–6. doi: 10.1001/jama.2013.6871 23821094 PMC4903171

[11] Mansournia MA, Altman DG. Inverse probability weighting. Bmj. 2016;352:i189. doi: 10.1136/bmj.i189 26773001

[12] Robins J. A new approach to causal inference in mortality studies with a sustained exposure period—application to control of the healthy worker survivor effect. Mathematical Modelling. 1986;7(9–12):1393–512.

[13] Robins JM, Greenland S, Hu F-C. Estimation of the causal effect of a time-varying exposure on the marginal mean of a repeated binary outcome. Journal of the American Statistical Association. 1999;94(447):687–700.

[14] Mansournia MA, Etminan M, Danaei G, Kaufman JS, Collins G. Handling time varying confounding in observational research. bmj. 2017;359:j4587. doi: 10.1136/bmj.j4587 29038130

[15] Mokhayeri Y, Hashemi-Nazari SS, Khodakarim S, Safiri S, Mansournia N, Mansournia MA, et al. Effects of Hypothetical Interventions on Ischemic Stroke Using Parametric G-Formula. Stroke. 2019;50(11):3286–8. doi: 10.1161/STROKEAHA.119.025749 31480969

[16] Shakiba M, Mansournia MA, Salari A, Soori H, Mansournia N, Kaufman JS. Accounting for time-varying confounding in the relation between obesity and coronary heart disease: Analysis with G-estimation, the Atherosclerosis Risk in Communities (ARIC) study. American journal of epidemiology. 2017. 10.1093/aje/kwx360 PMC5982707 29155924

[17] Aryaie M, Sharifi H, Saber A, Nazemipour M, Mansournia MA. Longitudinal Causal Effects of Normalized Protein Catabolic Rate on All-Cause Mortality in Patients with End-Stage Renal Disease: Adjusting for Time-varying Confounders Using G-estimation Method. Am J Epidemiol. 2020. 10.1093/aje/kwaa281 33350437

[18] Naimi AI, Cole SR, Kennedy EH. An introduction to g methods. International journal of epidemiology. 2017;46(2):756–62. doi: 10.1093/ije/dyw323 28039382 PMC6074945

[19] Vansteelandt S. Estimating direct effects in cohort and case–control studies. Epidemiology. 2009:851–60. doi: 10.1097/EDE.0b013e3181b6f4c9 19806060

[20] Chen A, Ilya S, Judea P, editors. Identifiability of Path-Specific Effects. International Joint Conference on Artificial Intelligence; 2005 August 2005; Edinburgh, Schotland.

[21] VanderWeele TJ, Tchetgen Tchetgen EJ. Mediation analysis with time varying exposures and mediators. Journal of the Royal Statistical Society: Series B (Statistical Methodology). 2017;79(3):917–38. doi: 10.1111/rssb.12194 28824285 PMC5560424

[22] Lin S-H, Young J, Logan R, Tchetgen EJT, VanderWeele TJ. Parametric mediational g-formula approach to mediation analysis with time-varying exposures, mediators, and confounders. Epidemiology (Cambridge, Mass). 2017;28(2):266–74. 10.1097/EDE.0000000000000609 PMC5285457 27984420

[23] Bild DE, Bluemke DA, Burke GL, Detrano R, Diez Roux AV, Folsom AR, et al. Multi-ethnic study of atherosclerosis: objectives and design. American journal of epidemiology. 2002;156(9):871–81. doi: 10.1093/aje/kwf113 12397006

[24] Nettleton JA, Schulze MB, Jiang R, Jenny NS, Burke GL, Jacobs DR Jr. A priori–defined dietary patterns and markers of cardiovascular disease risk in the Multi-Ethnic Study of Atherosclerosis (MESA). The American journal of clinical nutrition. 2008;88(1):185–94. doi: 10.1093/ajcn/88.1.185 18614740 PMC2504029

[25] Pearl J. The causal mediation formula—a guide to the assessment of pathways and mechanisms. Prevention science. 2012;13(4):426–36. doi: 10.1007/s11121-011-0270-1 22419385

[26] Etminan M, Collins GS, Mansournia MA. Using causal diagrams to improve the design and interpretation of medical research. Chest. 2020;158(1):S21–S8. doi: 10.1016/j.chest.2020.03.011 32658648

[27] Mansournia MA, Higgins JP, Sterne JA, Hernán MA. Biases in randomized trials: a conversation between trialists and epidemiologists. Epidemiology (Cambridge, Mass). 2017;28(1):54. doi: 10.1097/EDE.0000000000000564 27748683 PMC5130591

[28] Mansournia MA, Collins GS, Nielsen RO, Nazemipour M, Jewell NP, Altman DG, et al. A CHecklist for statistical Assessment of Medical Papers (the CHAMP statement): explanation and elaboration. Br J Sports Med. 2021;55(18):1009–17. doi: 10.1136/bjsports-2020-103652 33514558 PMC9110112

[29] Etminan M, Nazemipour M, Candidate MS, Mansournia MA. Potential Biases in Studies of Acid-Suppressing Drugs and COVID-19 Infection. Gastroenterology. 2021;160(5):1443–6. doi: 10.1053/j.gastro.2020.11.053 33340538 PMC7832226

[30] Etminan M, Brophy JM, Collins G, Nazemipour M, Mansournia MA. To Adjust or Not to Adjust: The Role of Different Covariates in Cardiovascular Observational Studies. Am Heart J. 2021;237:62–7. doi: 10.1016/j.ahj.2021.03.008 33722586

[31] Mansournia MA, Nazemipour M, Etminan M. Causal diagrams for immortal time bias. Int J Epidemiol. 2021. doi: 10.1093/ije/dyab157 34333642

[32] Mansournia MA, Hernán MA, Greenland S. Matched designs and causal diagrams. Int J Epidemiol. 2013;42(3):860–9. doi: 10.1093/ije/dyt083 23918854 PMC3733703

[33] Metropolis N, Ulam S. The monte carlo method. Journal of the American statistical association. 1949;44(247):335–41. doi: 10.1080/01621459.1949.10483310 18139350

[34] Clair C, Rigotti NA, Porneala B, Fox CS, D’Agostino RB, Pencina MJ, et al. Association of smoking cessation and weight change with cardiovascular disease among adults with and without diabetes. Jama. 2013;309(10):1014–21. doi: 10.1001/jama.2013.1644 23483176 PMC3791107

[35] Feodoroff M, Harjutsalo V, Forsblom C, Groop P-H, Group FS. Dose-dependent effect of smoking on risk of coronary heart disease, heart failure and stroke in individuals with type 1 diabetes. Diabetologia. 2018;61(12):2580–9. doi: 10.1007/s00125-018-4725-9 30229273

[36] Doyle JT, Heslin AS, Hilleboe HE, Formel PF. Early diagnosis of ischemic heart disease. New England Journal of Medicine. 1959;261(22):1096–101. doi: 10.1056/NEJM195911262612202 13817935

[37] Woodward M, Moohan M, Tunstall-Pedoe H. Self-reported smoking, cigarette yields and inhalation biochemistry related to the incidence of coronary heart disease: results from the Scottish Heart Health Study. Journal of epidemiology and biostatistics. 1999;4(4):285–95. 10764242

[38] Mucha L, Stephenson J, Morandi N, Dirani R. Meta-analysis of disease risk associated with smoking, by gender and intensity of smoking. Gender medicine. 2006;3(4):279–91. doi: 10.1016/s1550-8579(06)80216-0 17582369

[39] Warner KE. Possible increases in the underreporting of cigarette consumption. Journal of the American Statistical Association. 1978;73(362):314–8.

[40] Mansournia MA, Danaei G, Forouzanfar MH, et al. Effect of physical activity on functional performance and knee pain in patients with osteoarthritis: analysis with marginal structural models. Epidemiology. 2012:631–40. doi: 10.1097/EDE.0b013e31824cc1c3 22415107

[41] Robins JM, Wasserman L, editors. Estimation of effects of sequential treatments by reparameterizing directed acyclic graphs. Proceedings of the Thirteenth conference on Uncertainty in artificial intelligence; 1997: Morgan Kaufmann Publishers Inc.

[42] Schomaker M and Heumann C. Bootstrap inference when using multiple imputation. Statistics in Medicine. 2018;37(14):2252–66. doi: 10.1002/sim.7654 29682776 PMC5986623

