# Supplementary material for: Does weight mediate the effect of smoking on coronary heart disease? Parametric mediational g-formula analysis
Source: PLoS One. 2022 Jan 13;17(1):e0262403. doi: 10.1371/journal.pone.0262403 (PMC8757910; doi:10.1371/journal.pone.0262403)
Supplement: S1 Appendix — (PDF) [file pone.0262403.s001.pdf]

APPENDIX 1

mGFORMULA macro (mediational g-formula)

```
%let mycov=

fixedcov= age sex race cigcat income education alcohol diet,

       ncov=7,

cov1 = pa_intentional,      cov1otype = 4,          cov1ptype = lag1qdc,
cov2 = htn,                 cov2otype = 2,          cov2ptype = lag1bin,
cov3 = htnmed,              cov3otype = 1,          cov3ptype = lag1bin,
cov4 = chol,                cov4otype = 3,          cov4ptype = lag1qdc,
cov5 = aspirin,             cov5otype = 2,          cov5ptype = lag1bin,
cov6 = weight,              cov6otype = 3,          cov6ptype = lag1qdc,
cov7 = cigsday,             cov7otype = 4,          cov7ptype = lag1qdc,

       seed= 7834;

%mgformula (datain=mydata.med_final, id =mesaid , time=time , timepoints=5,
yvar=outcome , yreg=binsurv,  avar=cigsday , mvar=weight, a1value=20,
a0value=0,  nboot  =500);
```

G-formula macro

```
%let interv1 = intno=1, nintvar=1,
intlabel= ' cig=0',
intvar1= cigsday, inttype1 = 1, intvalue1=0, inttimes1 = 0 1 2 3 4;

%let interv2 = intno=2, nintvar=1,
intlabel= 'cig=20',
intvar1= cigsday, inttype1 = 1, intvalue1=20, inttimes1 = 0 1 2 3 4;


**GFORMULA Call;
title 'GFORMULA SAMPLE';
%gformula(
data= mydata.med_final,
id=mesaid,
time=time,
timepoints = 5,
outc=outcome,
outctype=binsurv,
fixedcov= age sex race cigcat income education alcohol diet,
timeptype= concat,
timeknots = 1 2 3 4,

ncov=7,

cov1 = pa_intentional,      cov1otype = 4,          cov1ptype = lag1qdc,
cov2 = htn,                 cov2otype = 2,          cov2ptype = lag1bin,
cov3 = htnmed,              cov3otype = 1,          cov3ptype = lag1bin,
cov4 = chol,                cov4otype = 3,          cov4ptype = lag1qdc,
cov5 = aspirin,             cov5otype = 2,          cov5ptype = lag1bin,
cov6 = weight,              cov6otype = 3,          cov6ptype = lag1qdc,
cov7 = cigsday,             cov7otype = 4,          cov7ptype = lag1qdc,

seed= 7834, nsamples =500, refint=1, numint=2
);
```
